# The tobacco industry at the health–environment nexus: a framing analysis of the UK ‘disposable’ e-cigarette ban

**DOI:** 10.1093/heapro/daaf218

**Published:** 2025-12-23

**Authors:** Pornpichar Lomngam, Rosemary Hiscock, Karen Evans-Reeves, Britta K Matthes

**Affiliations:** Department of Social and Policy Sciences, University of Bath, Claverton Down, Bath BA2 7AY, United Kingdom; Department for Health, University of Bath, Claverton Down, Bath BA2 7AY, United Kingdom; Department for Health, University of Bath, Claverton Down, Bath BA2 7AY, United Kingdom; Department for Health, University of Bath, Claverton Down, Bath BA2 7AY, United Kingdom

## Abstract

The sharp rise in ‘disposable’, or single-use, e-cigarettes in the UK—especially among young people—has raised urgent public health and environmental concerns. Manufacturers, including transnational tobacco companies (TTCs), have faced criticism for designing and marketing products that appeal to youth and have a considerable environmental footprint. In response, a UK-wide ban on single-use e-cigarettes was implemented in June 2025. While industry resistance to health policy is well documented, this is the first study to examine how TTCs and TTC-linked actors responded to regulation framed around both health and environmental objectives. We analysed 21 submissions to three public consultations from four TTCs and 10 TTC-linked organizations. Drawing on a taxonomy of framing strategies from an evidence-based corporate political activity framework, we explored how these actors sought to shape policymakers’ perceptions of the problem and its solutions. TTCs and TTC-linked organizations positioned themselves as responsible actors aligned with public health and environmental goals while opposing the ban. Youth use and environmental harms were reframed as problems of individual noncompliance and enforcement failure. The ban was portrayed as disproportionate, procedurally flawed, economically harmful, and likely to increase illicit trade and tobacco use. Industry-preferred alternatives included measures targeting individual behaviour and product innovation. These findings add to evidence of a disconnect between TTCs’ claimed transformation and their continued resistance to regulation. Close collaboration between public health and environmental advocates is needed to pre-empt and counter industry framing and influence, and to advance regulation that promotes public and planetary health.

Contribution to Health Promotion

- Provides the first analysis of the tobacco industry’s response to a proposed single-use e-cigarette ban, justified on both public health and environmental grounds
- Uses a framing analysis to reveal how transnational tobacco companies position themselves as responsible actors while resisting regulation
- Highlights the gap between industry ‘transformation’ narratives and policy behaviour, especially regarding youth and environmental protection
- Supports anticipatory, cross-sector policy approaches to pre-empt and counter industry influence and promote integrated action on human and planetary health

## BACKGROUND

Since the late 2010s, the use of electronic cigarettes (e-cigarettes; commonly known as vapes) among British youth has increased. By 2023, one in five 11–17-year-olds had tried e-cigarettes, and single-use (‘disposable’) devices became the dominant type, rising from 7% in 2019 to 69% in 2023 (ASH 2025). E-cigarettes create nicotine dependence in those who have never smoked, and although long-term health effects remain uncertain, evidence links them to lung injury, poisoning, burns from malfunctioning devices, and adverse mental health outcomes (Banks *et al.* 2023, Golder *et al.* 2025). Young e-cigarette users are also more likely than nonusers to start cigarette smoking (Golder *et al.* 2025). In addition, the environmental impact of single-use e-cigarettes is severe (see Table 1).

**Table 1.** Environmental impact of single-use e-cigarettes.

The design of single-use e-cigarettes generates waste, including critical raw materials such as lithium and copper. Each year, the lithium discarded in UK single-use e-cigarettes alone could power more than 10 000 electric vehicles (Material Focus 2025). Most e-cigarettes are discarded in household waste or general recycling (Truth Initiative 2021, Material Focus 2025), leading to the release of toxic chemicals and heavy metals (Ngambo *et al.* 2023) and fires and explosions from batteries (Pourchez *et al.* 2022). Although most constituent materials could be recovered when e-cigarettes are disposed of via electronic waste collection points or retailer take-back schemes, separation of materials is difficult and facilities are not always available (Pourchez *et al.* 2022, Deegan 2024). The plastic casing remains largely unrecyclable due to contamination from embedded nicotine residue, mixed materials, and the difficulty of separating components (Pourchez *et al.* 2022, Material Focus 2025).

Citing youth and environmental protection concerns, and following the ‘Creating a smokefree generation and tackling youth vaping consultation’, the UK government announced in early 2024 a ban on the sale, supply, and distribution of single-use e-cigarettes (UK Government 2024a). Using powers under the 1990 Environmental Protection Act, a UK-wide ban came into force on 1 June 2025 (Defra 2025). The devolved nations implemented it through their respective legislative or regulatory instruments, and in Scotland, this followed two consultations. The ban aligns the UK with jurisdictions such as Australia, Belgium, France, and some Swiss cantons (Chini 2024, Das 2024, Davey 2024).

The ban posed potential revenue losses for companies involved in the production and sale of single-use e-cigarettes, including the four major transnational tobacco companies (TTCs) (Hiscock *et al.* 2023). TTCs remain among the largest corporations globally (Murphy and Schifrin 2025) and continue to sell billions of cigarettes while expanding their product portfolios. They capitalized on the rapidly growing e-cigarette market by launching their own products as well as acquiring companies selling such products (TobaccoTactics 2024). Although Chinese manufacturers dominate, TTCs held 14% of the UK e-cigarette market in 2024 (Euromonitor International 2025).

TTCs are working to (re)position themselves as responsible actors in the environmental and public health spheres by adopting environmentally focused corporate social responsibility (TobaccoTactics 2022), ecological marketing (Houghton *et al.* 2018), and by promoting a ‘transformation’ narrative centred on products they describe as ‘reduced risk’ like e-cigarettes (Hill *et al.* 2023). Presenting themselves as responsible providers of such products, they seek to gain access to and shape policymaking (Hawkins and van Schalkwyk 2025).

However, evidence casts doubt on claims that TTCs are undergoing genuine transformation to reduce deaths and disease caused by their products: cigarette shipment decline is slow (Mehegan *et al.* 2024), young people remain heavily exposed to industry advertising—especially on social media (Cochran *et al.* 2023, Watts *et al.* 2024, Hardie *et al.* 2025b )—and TTCs actively oppose measures aimed at reducing e-cigarette youth uptake such as display, flavour, and packaging restrictions or stringent licensing schemes (Hardie *et al.* 2023, Matthes *et al.* 2025).

These strategies broadly align with those used by other commercial actors whose products and practices harm public and planetary health, yet who seek to position themselves as legitimate policy actors to resist regulation that threatens profits (Gilmore *et al.* 2023). For example, fossil fuel companies use greenwashing to appear as environmental stewards while derailing regulation that aims to protect the environment (Smerecnik and Renegar 2010, Cherry and Sneirson 2012, Plec and Pettenger 2012, Nurse 2015). In the case of the tobacco industry, no study to date has examined how it positions itself in response to policies justified on environmental grounds.

This paper is the first to analyse the tobacco industry’s response to a single-use e-cigarette ban. It examines the framing strategies used by TTCs and TTC-linked actors to influence UK policymakers’ perceptions of the problem and its solutions.

## METHODS

### Data collection

We collected responses to three consultations: the UK-wide ‘Creating a smokefree generation and tackling youth vaping consultation’ (UK Government 2024b), ‘Draft Environmental Protection (Single-use Vapes) (Scotland) Regulations 2024’ (Scottish Government 2024a), and ‘Implementing the prohibition of the sale and supply of single-use vapes in Scotland’ (Scottish Government 2024b).

Responses to the UK-wide consultation were not publicly available. We obtained submissions from a journalist who had made a Freedom of Information request to the Department of Health and Social Care (DHSC) for responses from 13 named organizations. Four of this consultation's 71 questions concerned single-use e-cigarettes. For the Scottish consultations, 111 submissions were available and downloaded. In the first consultation, two of five questions addressed the ban; in the second, 12 of 20.

### Data analysis

Potential connections between consultation respondents and TTCs were assessed via searches of the TobaccoTactics.org website. Submissions without known links to TTCs were excluded. We grouped actors by key function (manufacturer, trade association, think tank, and lobbying organization).

To analyse framing strategies, we employed a taxonomy from a corporate political activity framework, developed from the conceptual literature across unhealthy commodity industries (Ulucanlar *et al.* 2023), previously applied to a similar dataset of industry submissions (Matthes *et al.* 2025). The taxonomy outlines framing strategies commonly used to contest regulation, categorizing how corporate actors construct ‘good versus bad’ narratives to shape interpretations of key issues—actors, problems, and solutions.

Data were coded using NVivo 14 (Lumivero). The taxonomy was modified as needed to add or adapt categories; subcategories were identified inductively. Two coders (P.L. and B.K.M.) pilot-coded 25% of submissions; P.L. coded the remainder, cross-checked by B.K.M.

This project received approval from the University of Bath’s Biomedical Sciences Research Ethics Committee (reference 11492-13225).

## RESULTS

### Sample

We included 21 submissions from 14 actors: nine to the UK-wide consultation, eight to the first, and four to the second Scottish consultation. Two actors submitted to all three consultations, and two submitted to two.

The 14 actors were four TTCs (manufacturers), four trade associations, four lobbying organizations, and two think tanks (see Supplementary Table S1).

### Framing strategies

We present the key framing strategies by frame (actors/problems/solutions) and by frame-supporting claim (in italics). An overview is provided in Table 2.

**Table 2.** Framing strategies regarding the ban on the sale and supply of single-use e-cigarettes [*claims and insertions in italics were not part of the original framework (Ulucanlar *et al*. 2023)].

Framing the policy space	Frame-supporting claims*	Key illustrations and examples
The ‘good’ actor(s)	**We are** **legitimate policy actors**	**We understand the need to address the issue:** ‘[T]he use of disposable vapes by children is an issue that should be addressed’. (smokers’ rights group) ‘We recognize the sustainability challenges associated with disposable products’. (TTC) **We want to be part of the discussion and collaborate with the government**: ‘We are committed to actively participating in these crucial environmental and public health discussions’. (trade association)
	**We are** **legitimate scientific actors**	**We have relevant data and expertise:** ‘Our own consumer polling indicates…’. (trade association) ‘Our research shows …’. (TTC) **We support evidence-based policy**: ‘We also want smart, evidence-based regulations in place […] to make smoke-free a reality in the UK’. (TTC)
	**We are** **champions of public health**	**We are committed to public health goals**: ‘We have a zero-tolerance approach to youth access and use’. (TTC) **We only target adults who smoke/use nicotine**: ‘All our vape products focus solely on meeting the expectations of existing adult smokers and nicotine users’. (TTC) **We engage in initiatives supporting public health goals**: ‘[Our] campaign aims to send a clear message that if you smoke and wish to quit, then consider vaping, but if you don’t smoke, don’t vape’. (trade association)
	***We are environmentally responsible***	**We are committed to environmental goals**: ‘[We are] known for being a retail organization supportive of well-judged measures to deter litter and preserve the earth's precious resources’. (trade association) **We have taken action to make our products more environmentally friendly**: ‘[We] have made disposable devices easier to recycle by investing in technology such as removable batteries, which helps reduce the likelihood of plastics and batteries becoming mixed in household waste streams’. (TTC) **We have taken measures to support correct disposal**: ‘[We] offer a “take-back” solution (…) through the website’. (TTC) ‘[We] also launched a campaign […], with members, to provide 1000 free vape recycling bins to convenience retailers’. (trade association) ‘[O]ur on-pack QR codes guiding consumers on how to dispose of vapes safely’. (TTC)
The ‘bad’ actor(s)	**Policymakers *and relevant authorities* have questionable skills and motives**	**The government rushed the decision and overlooked critical issues**: ‘[T]he Scottish Government [has a] hurried approach to the policymaking process’. (trade association) ‘The Scottish Government has not fully considered how innovations such as this could improve the sustainability of disposable vapes’. (TTC) **The enforcement agency needs to improve:** ‘Trading Standards [needs] to get its act together’. (think tank)
The problem(s)	***Health and environmental harms arise from activities of other actors***	**The illicit market is the problem:** ‘[T]his illicit market already exists and accounts for the majority of illegal products on the market and being sold to children’. (trade association) **Products from irresponsible manufacturers are the problem:** ‘We are concerned by the availability of a large number of disposable vapes that, in addition to having irresponsible packaging or design, do not comply with the law’. (TTC) **Irresponsible sellers are the problem:** ‘It is clear that some owners and untrained staff in outlets such as cafes and tanning salons have a hazy or casual approach to the law… rogue traders on the High Street, in addition to black market sellers on social media, deliver direct to homes or street corners near school gates’. (trade association) **The lack of enforcement is the problem:** ‘Trading Standards presently do not have the resources to properly enforce the current regulation and that has created the situation leading to the problems highlighted’. (trade association)
	***Newer products are not a problem***	**Single-use e-cigarettes are crucial for smoking cessation:** ‘[D]isposable vapes provide adult smokers with a convenient and accessible format that can accelerate the reduction in smoking rates’. (TTC) **Single-use e-cigarettes don’t cause youth use:** ‘Youth vaping initiation is a consequence of tobacco use and other factors, rather than of the existence of disposable vapes’. (lobbying organization)
The ‘bad’ solution	**Policy is unnecessary and unacceptable**	**Sale of e-cigarettes to minors is already banned and needs to be better enforced:** ‘There is no point enacting further regulation when current regulations are being widely flouted’. (think tank), **The ban is disproportionate to the problem:** ‘[The ban is a] draconian measure’. (TTC) **The ban is unreasonable:** ‘Banning a product because it is sometimes consumed by people who are already banned from buying it is a poor basis for legislation. We do not ban cider just because some teenagers drink it. We do not ban 18 certificate films because some teenagers watch them. We do not even ban cigarettes because some teenagers smoke them’. (think tank) **The ban is unfair:** ‘[C]ompliant businesses that are meeting all their requirements being targeted for tighter restrictions even though they are already part of the ongoing solution’. (trade association)
	**Policy formulation contravenes norms, rules, and laws**	**The short consultation undermined effective stakeholder engagement:** ‘We share our sector colleagues’ concerns that the short period of consultation is not cohesive to good policy development and meaningful engagement with industry’. (trade association) **The government did not provide a (comprehensive) impact assessment:** ‘We are very seriously concerned that the disposable ban is being pursued without a published Impact Assessment, which takes full account of how adult vapers will respond to the disposable ban’. (TTC) ‘We note the lack of a business impact assessment alongside the publication of these regulations’. (trade association) **The ban will impede rights of businesses and users:** ‘An outright ban would disproportionally restrict the property rights of manufacturers and retailers and consumers’ rights of privacy’. (TTC) **The ban will violate trade agreements:** ‘A ban […] would violate International Trade Agreements’. (TTC)
	**Policy will lead to losses for businesses and economy**	**The ban will negatively affect retailers**: ‘It will cost legitimate Scottish businesses £51m in lost sales and millions more in the value associated with footfall loss, threatening the viability of some stores’. (trade association) **The ban will be costly for the state:** ‘[This] regulation would (…) increase burden on law enforcement’. (lobbying organization) **The ban will lead to losses for communities:** ‘From post office facilities, bill payment services and access to cash to deli counters, coffee and collection lockers, c-stores are designed to offer a wide range of services to their communities. A more restrictive range […], could encourage customers to […] reduce both footfall and business viability. Potentially putting all these services at risk’. (trade association) ‘Reducing footfall and total basket sales through convenience stores, and in turn impacting the wealth of the local community, local jobs and sales in the upstream supply chain’. (trade association)
	**Policy will fail and have perverse consequences**	**The ban will not work, and similar measures haven’t worked elsewhere:** ‘Just as with the failed War on Drugs, making products illegal does not make them disappear, but instead fuels crime to meet latent demand’. (think tank) **The ban will lead to an increase in illicit trade, and linked actors will benefit**: ‘The disposable vapes ban will lead to more customers […] moving to the already burgeoning illicit vape market’. (trade association) ‘Criminal gangs and other illegal traders would profit enormously from a ban on disposable vapes’. (lobbying organization) **The ban will lead to an increase in smoking and a decrease in cessation:** ‘This regulation would encourage those who were using disposable vapes back into cigarettes as they are perfect substitutes’. (lobbying organization) ‘A ban on single-use vapes, which are more affordable than re-usable vaping products, may deter many smokers from moving away from tobacco’. (trade association) **The ban will lead to confusion:** ‘We envisage a scenario of confusions for both retailers and consumers and enforcement agencies without resources to provide clarity’. (trade association) **The ban will pose risks to people, including minors, and the environment:** ‘By restricting the sale and supply, the government risks creating a large black market of illegal and potentially dangerous products’. (think tank) ‘There is also less control as to the quality and safety of the products being used, which is threat to the public and to the environment’. (trade association), **Ban will further inequities**: ‘[T]here is a concern that a ban on disposable vapes would disproportionately impact certain segments of the population, including vulnerable communities and individuals with limited access to other smoking cessation resources […], exacerbating existing health inequities’. (lobbying organization)
The ‘good’ solution(s)	**Solution is to help individuals to change their behaviours**	**Education**: ‘Instead of an outright ban, we recommend a more nuanced approach that focuses on targeted regulation and education initiatives to address underage vaping’ (lobbying organization), ‘…enhanced education regarding the necessity for the safe and environmentally friendly disposal of disposable vapes’. (trade association) **Sanctions to address youth use:** ‘We support fixed penalty notices for anyone caught importing or selling illegal vapes’. (TTC) ‘In considering producer obligations (…), we want to stress the importance of sanctions against irresponsible, free-riding producers of illicit, noncompliant vapes (making up a significant percentage of vapes present in the UK market) and their agents’. (TTC) **Stricter control measures to address youth use and illicit market:** ‘What is needed is firstly the introduction of a licence similar to the ones needed to sell alcohol and tobacco’. (trade association) ‘We recommend a more nuanced approach… [t]his could include stricter age verification measures for purchasing vape products’ (lobbying organization), ‘..,creation of a National Test Purchasing Scheme—ensuring retailers are held to account, with a requirement that they need to pass the test purchase scheme to retain their licence and providing that retailers can lose their licence if they are caught selling illegal vapes’. (TTC). ‘Extend the track and trace system that is currently applied for tobacco products to vaping products, providing for greater point of entry authentication and supply chain oversight’. (TTC) **Encourage proper disposal through improved recycling infrastructure**: ‘While there are environmental issues with how such devices are disposed of, that is better dealt with through the normal range of litter-avoidance schemes, from specific receptacles to device-return schemes’ (lobbying organization), ‘a deposit return scheme (…) to increase recyclability and improved recycling infrastructure’. (TTC)
	**Solution should not disrupt *TTCs’* business**	**More investment in enforcement and recycling infrastructure**: ‘Black market trading is significant but less visible—requiring adequate resources for detection and prosecution’. (trade association) ‘Increasing funding/strengthening capacity for the UK Border Force and Trading Standards to remove noncompliant products and impose stricter penalties’. (TTC) ‘Perhaps the [regulation] will need to include some funding for specific vape disposal containers’. (lobbying organization) **Collaboration with Chinese authorities**: ‘The government should ensure that it is building the necessary communications routes with the Chinese authorities to exploit this option for clamping down on illicit products’. (lobbying organization) **Self-regulation to address youth use:** ‘[We are] of the view that manufacturers and suppliers should rename and redesign the packaging of products to make them less appealing to children and young people’. (trade association) **Product and design innovation**: ‘Improved less-damaging designs’. (trade association) ‘We want it to be mandatory for single-use vapes to have removable batteries to support responsible recycling’. (TTC)

#### The actors

***We are the ‘good’ actors***. TTCs and linked organizations positioned themselves as *legitimate policy actors* by claiming to acknowledge the issues at stake—youth use and environmental harm—and suggesting to government that they discuss and collaborate on solutions. A trade association called for ‘meaningful working with business’ and ‘urge[d] the government to continue to engage with industry’.

To construct *legitimacy as scientific actors*, several referenced proprietary research—such as expert reports and consumer surveys—to demonstrate their expertise, and expressed commitment to evidence-based policy.

They also presented themselves as *champions of public health*, supporting public health goals, stating to target only adults with their products, and pointing to their own initiatives in support of health goals.

Lastly, TTCs sought to appear as *environmentally responsible actors*, committed to sustainability, and citing actions such as introducing removable batteries, promoting correct disposal through on-pack quick-response (QR) codes and take-back schemes, and providing disposal bins.

***These are the ‘bad’ actors***. TTCs and some linked actors *criticized the government* for its ‘hurried approach’ (trade association) and for failing to account for issues such as the ‘wellbeing of the […] economy’ (trade association) or innovations that ‘could improve [product] sustainability’ (TTC). One think tank remarked that Trading Standards needs ‘to get its act together’.

#### The ‘problems’

Several actors argued that the *problems—youth vaping and environmental harms—stem from other actors*, particularly those operating within the illicit market. One stated that the illicit market ‘account[s] for the majority of illegal products […] being sold to children’ (trade association), while another argued producers of illicit products ‘have no incentive to ensure their products are environmentally friendly or recyclable’ (lobbying organization). They emphasized the role of producers of ‘poor quality, nonregulation products’ (lobbying organization) and sellers such as ‘rogue traders on the High Street [and] black-market sellers on social media, deliver[ing] direct to homes or street corners near school gates’ (trade association).

Enforcement failures to protect against illicit trade and sale to minors were highlighted, with one trade association stating that Trading Standards lacks the resources to ‘properly enforce current regulation’.

Rather than depicting them as a problem, TTCs and linked actors framed *single-use e-cigarettes as essential for smoking cessation.* One lobbying organization argued: ‘[b]eing able to buy something that works without fuss is far less daunting, and often more appealing’, and another one urged that ‘disposable vapes should be […] endorsed as one option for smoking cessation’ and that youth use is ‘a consequence of tobacco use and other factors’, not of single-use e-cigarettes.

#### The 'solutions'

***The ban as the ‘bad’ solution***. Many actors argued that the proposed ban was both *unacceptable and unnecessary*, suggesting that existing restrictions were sufficient but poorly enforced. The ban was described as ‘draconian’ (TTC) and disproportionate. A lobbying organization stated: ‘The electronic waste produced by toys is estimated to be 77 times that of vaping… yet their prohibition is not discussed’. A TTC argued that the ban would unfairly place undue burden on ‘responsible producers’.

It was also suggested that the ban *contravened legal norms*. Actors criticized the short consultation period or the absence of comprehensive impact assessments—highlighting the lack of a business impact assessment—as well as property and privacy rights and trade agreement violations.

Several actors argued the ban would lead to *losses for businesses, economy, and society*, warning it would ‘threaten the viability of some stores’ (trade association), ‘impact the wealth of the local community [and] local jobs and sales’ (trade association), and ‘increase the burden on law enforcement’ (lobbying organization).

Finally, both TTCs and linked actors argued that the ban would *fail and have perverse outcomes*. They predicted it would increase illicit trade—with ‘[c]riminal gangs and other illegal traders [profiting] enormously’ (lobbying organization)—and lead to relapse: ‘some adult vapers [would return] to tobacco products’ (trade association). Additional concerns included health and safety risks from ‘less regulated, unsafe products’ (lobbying organization) and increased ‘criminal involvement for organized and petty crime’ (think tank). The ban was also framed as likely to ‘exacerbate existing health inequities’ by disproportionately impacting ‘vulnerable communities and individuals with limited access to other smoking cessation resources’ (lobbying organization).

***The ‘good’ solutions***. TTCs and linked actors proposed that policies should *target specific individuals or subpopulations, not the general public*. Suggested measures included education campaigns, stricter penalties for noncompliant sellers, targeting ‘free-riding producers of illicit, noncompliant vapes […] and their agents’ (TTC). To address youth use, actors called for stricter age-verification systems, a retailer licensing scheme, and test-purchasing operations. For environmental concerns, they recommended improved recycling infrastructure, including deposit return schemes and more recycling bins.

Preferred solutions were also *nondisruptive for TTCs*. Actors urged the government to invest more in enforcement. A lobbying organization suggested engaging with Chinese authorities ‘for clamping down on illicit products’. Another lobbying organization proposed government funding for ‘specific vape disposal containers’. A few actors advocated for self-regulation, including ‘renam[ing] and redesign[ing] products to make them less appealing to children and young people’ (trade association) and developing ‘less-damaging designs’ (lobbying organization), such as removable batteries. Mandatory removable batteries were only proposed by one TTC.

In the Scottish consultations where the ban likely appeared unavoidable, actors asked for a ‘business-friendly approach to [its] implementation’ (trade association), a prolonged transition period and ‘phased approach’ (TTC). A lobbying organization proposed that the ban should be delayed by 3 years until April 2028 and only become effective ‘if the number of children vaping increases by 10%’.

## DISCUSSION

This study provides the first analysis of how TTCs and linked organizations responded to three consultations on the UK’s ban on single-use (‘disposable’) e-cigarettes. While previous research has extensively documented how the tobacco industry uses framing strategies to oppose and delay public health policy (Ulucanlar *et al.* 2016), our findings show how these strategies are being adapted and extended into environmental governance, providing new insight into TTCs’ evolving efforts to resist regulation. The findings also resonate with strategies in other sectors, such as fossil fuels, where companies claim environmental stewardship while obstructing environmental policies (Cherry and Sneirson 2012, Plec and Pettenger 2012, Gilmore *et al.* 2023).

With calls for an EU-wide single-use e-cigarette ban (Wilfer 2024) and some countries considering similar measures (Reuters 2024, ECigIntelligence 2025), alongside growing momentum to ban cigarette filters (ASH US 2024), it is crucial to understand how TTCs respond to policies at the health–environment nexus, so that effective, evidence-based regulation can be advanced.

As in other unhealthy commodity industries (Ulucanlar *et al.* 2023), TTCs and linked organizations portrayed themselves as legitimate and responsible policy contributors. While youth use and environmental harms were acknowledged, these issues were reframed as a matter of individual behaviour or enforcement failure—rather than of product design, availability, or marketing. In doing so, TTCs seek to reposition themselves not as the problem but as part of the solution, using narratives of responsibility to deflect accountability and health-/environment-wash their public image.

Arguments against the ban—framing it as disproportionate, procedurally flawed, economically harmful, and likely to increase illicit trade—echo industry rhetoric seen in responses to e-cigarette regulation (Hamilton *et al.* 2024, Harrison *et al.* 2024, Matthes *et al.* 2025) and tobacco control measures such as standardized packaging (Hatchard *et al.* 2014, Evans-Reeves *et al.* 2015, Evans-Reeves *et al.* 2019). Given the UK's early adoption, this opposition resembles earlier industry efforts to block policy and prevent precedents for wider diffusion (Hiilamo *et al.* 2014 , Crosbie *et al.* 2019).

Industry-preferred alternatives such as education campaigns, enforcement of existing laws, and voluntary recycling schemes shift attention from systemic to individual solutions and risk enabling less effective, industry-led regulation. These efforts resemble earlier strategies, including industry-funded youth prevention programmes that served to avoid regulation and failed to address youth smoking (Landman *et al.* 2002). Recycling schemes mirror industry-sponsored cigarette butt litter collection schemes, which TTCs use to present themselves as responsible actors while ignoring that they are the sole cause of the problem. These schemes may also risk delaying further regulation (e.g. filter ban) that would reduce environmental and health harms. Similar tactics have been observed in other sectors: e.g. the plastics industry has promoted anti-littering campaigns and personal responsibility messaging while resisting regulation (Lacy-Nichols *et al.* 2022), and fossil fuel companies have long pushed individual behavioural change (e.g. carbon footprint calculators) to deflect attention from their own environmental impacts (Bergan 2021).

As single-use e-cigarettes have been banned or severely restricted—as in New Zealand and the UK—observers have noted that old stock was sold off at discount prices while manufacturers introduced new reusable products (Evans-Reeves 2025, Hardie *et al.* 2025a, King 2025). These products look similar to their predecessors and are sold at a comparable price point, so consumers may have limited incentive to reuse them. Data from a leading waste firm show that 2 months after the UK ban, 3% more e-cigarettes were being incorrectly mixed with recycling than before the ban (King 2025).

This study highlights an opportunity for stronger public health and environmental collaboration. Recognizing shared goals can foster cross-sector coalitions to anticipate and counter industry interference, while anticipatory governance can pre-empt loopholes.

## CONCLUSION

This study shows how TTCs and TTC-linked actors used familiar framing strategies to oppose the UK’s single-use e-cigarette ban, despite claimed alignment with public health and environmental goals. Their resistance and attempts to deflect responsibility expose the gap between their ‘transformation’ rhetoric and their responses to potential and incoming regulation. As more countries consider similar measures, collaboration between public health and environmental actors—alongside anticipatory policy design—will be essential to counter industry influence and advance effective regulation that promotes both human and planetary health.

## Supplementary Material

daaf218_Supplementary_Data

## Data Availability

The responses to the Scottish consultations are publicly available. The responses to the UK-wide consultation, which we obtained through a journalist, are available upon reasonable request.
