# Supplementary material for: The tobacco industry at the health–environment nexus: a framing analysis of the UK ‘disposable’ e-cigarette ban
Source: Health Promot Int. 2025 Dec 23;40(6):daaf218. doi: 10.1093/heapro/daaf218 (PMC12721999; doi:10.1093/heapro/daaf218)
Supplement: daaf218_Supplementary_Data [file daaf218_supplementary_data.pdf]

Supplementary File

Table S1: Overview of actors and their connections to transnational tobacco companies (TTCs)

Actors	*Nature of known TTC link*	*Source*
**Manufacturers (TTC)**	n/a	(Tobacco Tactics, 2025a)
**Manufacturers (TTC)**	n/a	(Tobacco Tactics, 2025c)
**Manufacturers (TTC)**	n/a	(Tobacco Tactics, 2025d)
**Manufacturers (TTC)**	n/a	(Tobacco Tactics, 2025f)
**Trade association**	Membership	(Tobacco Tactics, 2024b)
**Trade association**	Membership	(Tobacco Tactics, 2020)
**Trade association**	Membership	(Tobacco Tactics, 2024f)
**Trade association**	Received funding from TTC	(Tobacco Tactics, 2024c)
**Think tank**	Received funding from TTCs	(Tobacco Tactics, 2024a)
**Think tank**	Received funding from TTCs	(Tobacco Tactics, 2024e)
**Lobbying organisation**	Received funding from TTCs	(Tobacco Tactics, 2024d)
**Lobbying organisation**	Received funding from TTCs	(Tobacco Tactics, 2025b)
**Lobbying organisation**	Set up by and funded by an organisation that received funding from TTCs, received funding from a TTC	(Tobacco Tactics, 2024g)
**Lobbying organisation**	Received funding from TTC-linked third party	(Tobacco Tactics, 2025e)

References

TOBACCO TACTICS. 2020. Scottish Wholesale Association. Available: https://www.tobaccotactics.org/article/scottish-wholesale-association/, accessed on 20/05/2025.

TOBACCO TACTICS. 2024a. Adam Smith Institute. Available: https://www.tobaccotactics.org/article/adam-smith-institute/, accessed on 20/05/2025.

TOBACCO TACTICS. 2024b. Association of Convenience Stores. Available: https://www.tobaccotactics.org/article/association-of-convenience-stores/, accessed on 20/05/2025.

TOBACCO TACTICS. 2024c. Federation of Independent Retailers (NFRN). Available: https://www.tobaccotactics.org/article/federation-of-independent-retailers-nfrn/, accessed on 20/05/2025.

TOBACCO TACTICS. 2024d. Forest. Available: https://www.tobaccotactics.org/article/forest/, accessed on 20/05/2025.

TOBACCO TACTICS. 2024e. Institute of Economic Affairs. Available: https://www.tobaccotactics.org/article/institute-of-economic-affairs/, accessed on 20/05/2025.

TOBACCO TACTICS. 2024f. Scottish Grocers' Federation. Available: https://www.tobaccotactics.org/article/scottish-grocers-federation/, accessed on 20/05/2025.

TOBACCO TACTICS. 2024g. World Vapers' Alliance. Available: https://www.tobaccotactics.org/article/world-vapers-alliance/, accessed on 20/05/2025.

TOBACCO TACTICS. 2025a. British American Tobacco. Available: https://www.tobaccotactics.org/article/british-american-tobacco/, accessed on 20/05/2025.

TOBACCO TACTICS. 2025b. Consumer Choice Center. Available: https://www.tobaccotactics.org/article/consumer-choice-center/, accessed on 20/05/2025.

TOBACCO TACTICS. 2025c. Imperial Brands. Available: https://www.tobaccotactics.org/article/imperial-brands/, accessed on 20/05/2025.

TOBACCO TACTICS. 2025d. Japan Tobacco International. Available: https://www.tobaccotactics.org/article/japan-tobacco-international/, accessed on 20/05/2025.

TOBACCO TACTICS. 2025e. Knowledge-Action-Change. Available: https://www.tobaccotactics.org/article/knowledge-action-change/, accessed on 20/05/2025.

TOBACCO TACTICS. 2025f. Philip Morris International. Available: https://www.tobaccotactics.org/article/philip-morris-international/, accessed on 20/05/2025.
